# Around the Hospitals

**Published:** 1893-07-01

**Authors:** 


### Around the Hospitals.

Notice to the Managers of Institutions.—Special space is now reserved for the insertion of notices of hospital meetings and festivals. The Editor requests that all notices may reach The Hospital Office, 428, Strand, at least one week before the date of each meeting.

**The Royal Free Hospital.**—The report of the Royal Free Hospital for 1892 shows an improvement in financial prosperity. The greatest increase is shown under the heading of legacies. An income that depends on legacies is, however, a most precarious one. Not counting on this source, the hospital has an expenditure of £10,551, or about double the dependable income of £5,119. In 1892 the legacies amounted to £8,864, leaving a balance on the average expenditure; but in 1891 legacies only reached £258, making the income very considerably less than the expenditure. It is, therefore, necessary to greatly increase the reliable income of the institution. We are glad to note that subscriptions have advanced. Donations, on the other hand, show a falling off. In the nursing department the gains to the hospital do not appear to be so great as in former years, but we trust that as the Trained Nurses' Institute maintains its high standard of efficiency and popularity, that the smaller returns to the hospital are due to increased comfort to the nursing staff. London owes a special debt of gratitude to the Royal Free Hospital. It was founded to meet the pressing want of the poor at a time when no other medical charity opened its doors for the immediate reception of the destitute sick, free of charge and without letters of recommendation. Since the year 1828 over two millions of the sick poor have found relief within its walls. This goodly record has received recognition during the past year by the grant of a Royal charter of incorporation. The hospital being urgently in need of improvements and repairs, some have already been affected, whilst the rebuilding of the front of the hospital is to be commenced shortly. Sufficient funds are not forthcoming to complete the work, but before the most necessary task is completed it is hoped and believed that friends will be found to provide the whole sum necessary.

**The Aberdeen Hospital for Sick Children.**—This hospital continues to receive an increasing number of patients, 601 cases having been treated during the year 1892. The expenditure shows a very moderate increase in proportion. A small debt still remains to be cleared off. The lady collectors have contributed £423 to the income, a very substantial help. The two convalescent homes prove themselves most useful, and form also a pleasant change for the nursing staff, who are transferred there for short periods in rotation.

**The Queen's Hospital, Birmingham.**—The report of this hospital for the past year shows that it continues to deservedly increase in popularity, and that the recent extensions were much needed. The extension has been mainly in the direction of beds for accidents, as the population to which the hospital ministers is especially liable to this form of calamity. Whilst the income shows a slight falling off, the expenditure has increased. The decrease is mainly visible in subscriptions and congregational collections. It is obvious that special efforts will have to be made to meet the increased demands of the larger hospital. As we mentioned on a previous occasion, a registration fee of one shilling is required of casualty and out-patients at this institution.

